# PET/CT-aided biopsy of lung lesions enhances diagnostic efficacy, especially for lesions >3cm

**DOI:** 10.3389/fonc.2024.1296553

**Published:** 2024-01-30

**Authors:** Wangzheng Liu, Bin Ji, Lin Bai, Shi Gao

**Affiliations:** Department of Nuclear Medicine, China-Japan Union Hospital of Jilin University, Changchun, China

**Keywords:** CT-guided transthoracic needle biopsy, PET/CT, ^18^F-fluorodeoxyglucose, lung cancer, PET/CT-guided biopsy

## Abstract

**Objectives:**

The purpose of this study was to compare the diagnostic efficacy of PET/CT-aided CT-guided and routine CT-guided transthoracic needle biopsy for lung lesions.

**Methods:**

A total of 458 patients with suspicious lung lesions were referred for CT-guided biopsy, with 227 patients assigned to the PET/CT group and 231 patients assigned to the CT group. The clinical characteristics and diagnostic yield were compared between the two groups. Furthermore, conducting subgroup analysis to evaluate the differences of diagnostic success or failure between the two groups.

**Results:**

The sensitivity and diagnostic accuracy rate differed significantly (P = 0.035, P = 0.048). In the PET/CT group, the values were 95.7% and 96.3%, respectively, while in the CT group, they were 90.1% and 91.9%. When considering non-diagnostic cases, the overall diagnostic success rate increased markedly in PET/CT group (93.0% vs. 83.1%, P = 0.001). In our subgroup analysis, the PET/CT group demonstrated superiority in detecting lesions larger than 3 cm (OR, 4.81; 95CI%, 2.03 - 11.36), while showing a moderate effect in lesions smaller than 3 cm (OR, 1.09; 95CI%, 0.42 - 2.81). Significant effect modification was observed in large lesions in the PET/CT group (P for interaction = 0.023).

**Conclusions:**

^18^F-FDG-PET/CT enhances the diagnostic efficacy of CT-guided transthoracic needle biopsy for lung lesions, and the incremental value can be modified by lesion size, particularly when the diameter is larger than 3 cm.

## 1 Introduction

Lung cancer is a prevalent malignant disease worldwide, leading to high mortality rates and affecting a significant number of individuals (1). When a suspicious lung lesion is detected, effective tissue sampling plays a crucial role in diagnosing and planning treatments for various subtypes of lung cancer, particularly for immunohistochemical analysis, molecular biology investigations, and gene detection (2). Over the years, CT-guided transthoracic needle biopsy (TTNB) has established itself as a dependable and secure diagnostic procedure for suspicious lung lesions (3). However, diagnostic failures are sometimes encountered in lung lesions due to factors such as fibrosis, necrosis, atelectasis, or obstructive pneumonia (4, 5).

Positron emission tomography/computed tomography (PET/CT) is capable of investigating the metabolic features of lung lesions and differentiating hypermetabolic areas that reflect the real biological behavior of the entire lesion (6). This capability helps to reduce the risk of diagnostic failure. Recently, PET/CT has been increasingly utilized in clinical practice for CT-guided needle biopsy, with studies highlighting its role in guiding target selection, elevating diagnostic precision, and reducing the need for repeated biopsies (7, 8). Furthermore, PET/CT-guided TTNB contribute to obtaining higher-quality samples for subsequent immunohistochemical and molecular biology investigations (9, 10).

Despite this, it is important to note that PET/CT has a high diagnostic performance of sensitivity and positive predictive value in patients with pulmonary nodules, especially those classified as intermediate and high-risk based on the Brock model (11). Conversely, patients with low metabolic nodules, which are often associated with benign lesions, are more likely to avoid biopsy. Consequently, many contrast studies have exhibited selection bias, with higher rates of malignant lesions in the PET/CT group (7, 12), which may serve as a potential confounding factor. Additionally, a study indicated PET/CT only provided an incremental value of 40.7% in lung biopsy (13). Considering the cost-effective of PET/CT examination, it becomes evident that identifying the population best suited for PET/CT-aided in lung biopsy is necessary. Therefore, the objective of this study was to compare the diagnostic efficacy of PET/CT-aided CT-guided TTNB with routine CT-guided TTNB for lung lesions and conduct subgroup analysis to explore variations between the two groups.

## 2 Materials and methods

### 2.1 Patient selection

Between January 2020 and August 2022, a total of 458 patients were referred for CT-guided TTNB. All patients were categorized into two groups based on whether they had previous PET/CT scans: the PET/CT group and the CT group. Among these patients, 227 underwent ^18^F-FDG PET/CT scans prior to the procedure, while 231 did not. The inclusion criteria were as follows (1): suspicion of a malignant lung lesion (2); confirmation of the final clinical diagnosis through surgical resection or at least 12 months of follow-up (3); patients in the PET/CT group were required to undergo PET/CT scans within a week before TTNB (4); in each group, only the first biopsy was evaluated for patients who underwent biopsies more than once. The exclusion criteria were as follows (1): immediate treatment with radiofrequency ablation or radiation for lung lesions diagnosed as malignant by biopsy (2); absence of subsequent radiological examination within 12 months for lung lesions diagnosed as benign by biopsy (3); patients who either passed away or were lost to follow-up prior to the final diagnosis.

### 2.2 ^18^F-FDG PET/CT imaging

The PET/CT scans were conducted using two dedicated diagnostic PET/CT devices: the uMI 510 unit and uMI 780 unit (United Imaging Healthcare, Shanghai, China). All PET/CT acquisitions were performed 40–60 minutes following intravenous injection of 3.5 MBq/kg of ^18^F-FDG after a fasting period of at least 6 hours. Integrated PET/CT images were corrected for scatter and attenuation based on CT information.

### 2.3 CT-guided biopsy

Prior to the biopsy, all patients received written informed consent. The international normalized ratio (INR) needed to be maintained below 1.5, and the minimum platelet counted maintained at 70000/μL. Impaired coagulation status and platelet counts needed to be corrected as much as possible if the above threshold are not met. Additionally, patients received instructions to discontinue anti-aggregant drugs 1-7 days before the procedure based on an individual risk-benefit assessment of medication cessation (14). All the planning and localization CT scans were conducted using the same 16-detector-row scanner (Symbia Intevo, Siemens Healthcare). For patients who underwent ^18^F-FDG PET/CT, the target for biopsy was determined by two experienced nuclear medicine physicians based on the analysis of previous PET/CT images visually. For patients who did not undergo ^18^F-FDG PET/CT, the parenchyma of lesions was chosen as the target for biopsy. CT-guided TTNB were performed by 3 nuclear medicine specialists who were trained to perform image-guided biopsies. Light sedation was administered as a preference. Following standard sterilization procedures, BioPince™ full core biopsy instrument (18G, Argon medical devices, INC) and Co-Axial introducer needle (MCXS1810BP, Argon medical devices, INC) were used to perform the CT-guided biopsy, resulting in the acquisition of 1 to 6 specimens. After removing needle, manual compression was applied to the puncture site for 2-3 minutes, and performed CT scans for entire lung immediately to rule out the presence of possible complications. The patients were then observed for at least 2 hours following the procedure to ensure hemodynamic stability and monitor their respiratory condition.

### 2.4 Pathology results of biopsy

The biopsy diagnoses were categorized into three groups: malignant, benign, and non-diagnostic (when only normal tissue or necrosis was found, or when the specimens obtained were inadequate for a conclusive histopathological evaluation). The final diagnosis were categorized as malignant and benign lesions through surgical resection or at least 12 months of follow-up. If malignant disease was detected in both the biopsy diagnosis and the final diagnosis, the biopsy results were considered as true positive (TP). True negative (TN) was confirmed if benign disease was detected in both the biopsy diagnosis and the final diagnosis. False positive (FP) indicates that the biopsy diagnosis was malignant, but the final diagnosis was benign, whereas false negative (FN) means that the biopsy diagnosis was benign, but the final diagnosis was malignant.

### 2.5 Statistical analysis

Normality was assessed by the Shapiro-Wilk test. The normal distribution variables were expressed as mean ± standard deviation (SD), while non-normal metric variables were expressed as median and interquartile range (IQR). Independent-sample t-tests, Mann-Whitney U-test, and chi-square test were used to analyze differences in clinical characteristics between the two groups. Sensitivity, specificity, positive predictive value (PPV), negative predictive value (NPV), and diagnostic accuracy rate were compared between the two groups by chi-square test. Further categorization was performed within each group, dividing them into two classes: the diagnostic success class (including TP and TN) and the diagnostic failure class (including cases of non-diagnostic, FP, and FN). Then, the potential confounding factors and effect modification were explored through subgroup analysis based on five criteria: final diagnosis (malignant vs. benign), lesion size (minimum diameter, ≤ 3 cm vs. > 3 cm), number of lesions (single vs. multiple), number of specimens (≤ 2 vs. ≥ 3), and Lobar location (upper or middle lobes vs. lower lobe). An odds ratio (OR) > 1.00 indicated a greater likelihood of diagnostic success in the PET/CT group. The Wald chi-square test were used to assess the interaction effect. Statistical significance was defined as a two-sided P-value < 0.05. All statistical analysis were completed using SPSS 25.0.

## 3 Results

The clinical characteristics of 458 patients were summarized in **Table 1**. Final diagnoses were achieved through surgical resection in 125 patients, other histopathological examinations or gene detection in 231 patients, and subsequent CT-images in 102 patients. According to the final diagnosis, 194 patients in the PET/CT group (194/227, 85.5%) were diagnosed as malignant, while 181 patients in the CT group (181/231, 78.4%) were diagnosed as malignant. Among the remaining 33 patients (33/227, 14.5%) in the PET/CT group were diagnosed as benign and 50 patients (50/231, 21.6%) in the CT group were diagnosed as benign. The distribution of malignant and benign lesions showed significant differences between the two groups (P = 0.048). There were no statistically significant differences in other variables between the two groups.

**Table 1:** Patient characteristic and differences between the two groups.

Characteristic	PET/CT group	CT group	P
Age (y)	64.2 (SD, 10.3)	63.5 (SD, 9.9)	0.470 ^t^
Sex			
Female	111 (48.9%)	94 (40.7%)	0.077 ^χ2^
Male	116 (51.1%)	137 (59.3%)	
Lesion size (cm)	4.1 (IQR, 2.9)	4.0 (IQR, 2.8)	0.331 ^m^
Number of lesions			
Single	154 (67.8%)	165 (71.4%)	0.404^χ2^
Multiple	73 (32.2%)	66 (28.6%)	
Number of specimens	3 (IQR, 1)	4 (IQR, 2)	0.453 ^m^
Lesion location			
LUL	53 (23.3%)	61 (26.4%)	0.743^χ2^
LLL	39 (17.2%)	47 (20.3%)	
RUL	72 (31.7%)	63 (27.3%)	
RML	11 (4.8%)	11 (4.8%)	
RLL	52 (22.9%)	49 (21.2%)	
Operators			
A	43 (18.9%)	31 (13.4%)	0.215^χ2^
B	176 (77.5%)	194 (84.0%)	
C	8 (3.5%)	6 (2.6%)	
Final diagnosis			
Malignant	194 (85.5%)	181 (78.4%)	0.048^χ2^
Benign	33 (14.5%)	50 (21.6%)	
Complications	33 (14.5%)	43(18.6%)	0.241^χ2^

Data are number and percentage, unless otherwise indicated.

^t^Independent-sample t-tests for normal distribution variables.

^t^Mann-Whitney U-test for non-normal metric variables.

^χ2^chi-square test for categoric variables.

LUL, left upper lobe; LLL, left lower lobe; RUL, right upper lobe; RML, right middle lobe; RLL, right lower lobe.

In the PET/CT group, there were 180 TP, 31 TN, and 8 FN. In the CT group, there were 154 TP, 38 TN, and 17 FN. Neither group had any FP results. The sensitivity, specificity, PPV, NPV, and diagnostic accuracy rate were 95.7%, 100%, 100%, 79.5%, and 96.3% in the PET/CT group respectively. The corresponding values were 90.1%, 100%, 100%, 69.1%, and 91.9% in the CT group (**Table 2**). Significant differences were observed in the sensitivity and diagnostic accuracy rate between the two groups (P = 0.035, P = 0.048). Resulting a lower false negative rate in the PET/CT group (8/188, 4.3% vs. 17/171, 9.9%).

**Table 2:** Diagnostic results and differences between the two groups.

Diagnostic results	PET/CT group	CT group	P
Sensitivity	95.7% (95%CI: 91.8% - 98.1%)	90.1% (95%CI: 84.6% - 94.1%)	0.035
specificity	100.0% (95%CI: 88.8% - 100.0%)	100.0% (95%CI: 90.7% - 100.0%)	>0.999
PPV	100.0% (95%CI: 98.0% - 100.0%)	100.0% (95%CI: 97.6% - 100.0%)	>0.999
NPV	79.5% (95%CI: 63.5% - 90.7%)	69.1% (95%CI: 55.2% - 80.9%)	0.261
Diagnostic accuracy rate	96.3% (95%CI: 92.9% - 98.4%)	91.9% (95%CI: 87.3% - 95.2%)	0.048

CI, Confidence interval.

**Figure 1:**
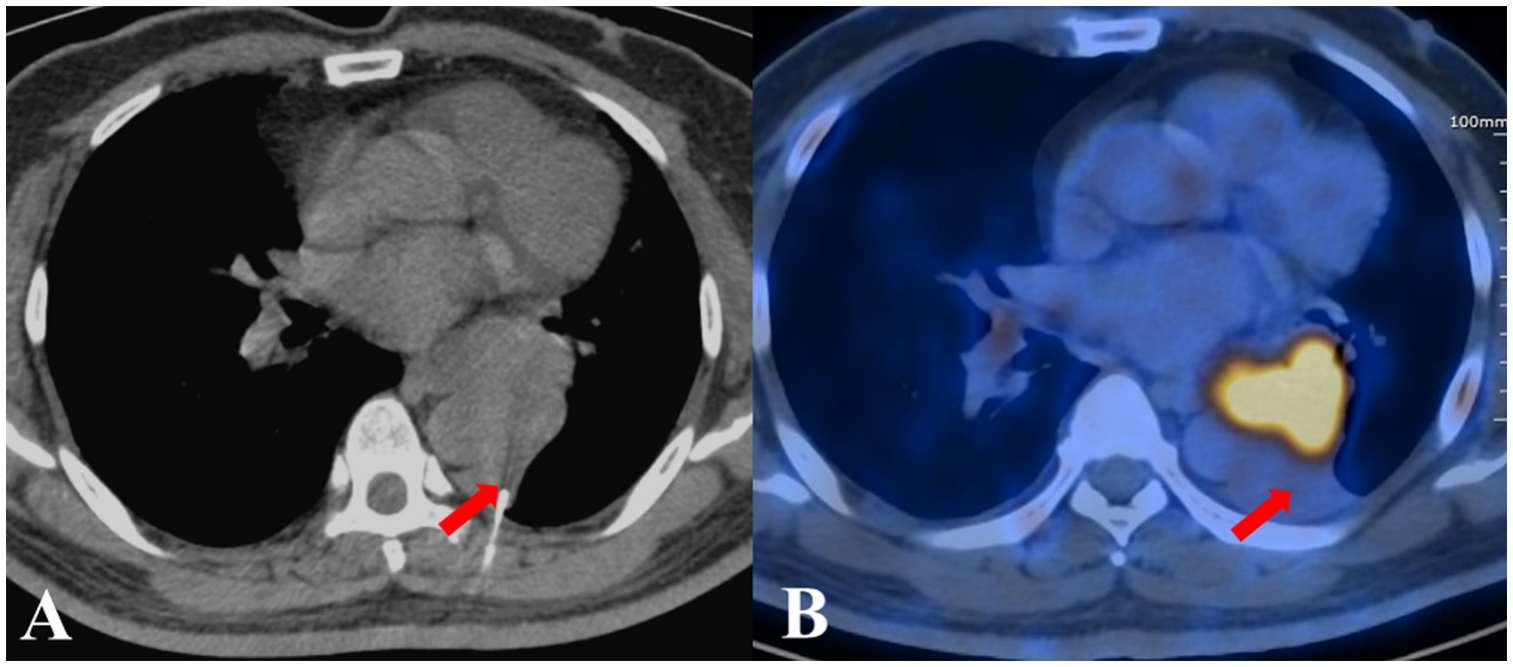
The biopsy pathological examination revealed foam cells and alveoli epithelial cells proliferation in a patient from the CT group, who was ultimately diagnosed with adenocarcinoma. **(A)** CT-guided TTNB. **(B)** After five days, PET/CT images showed the location of needle tip was hypometabolic.

**Figure 2:**
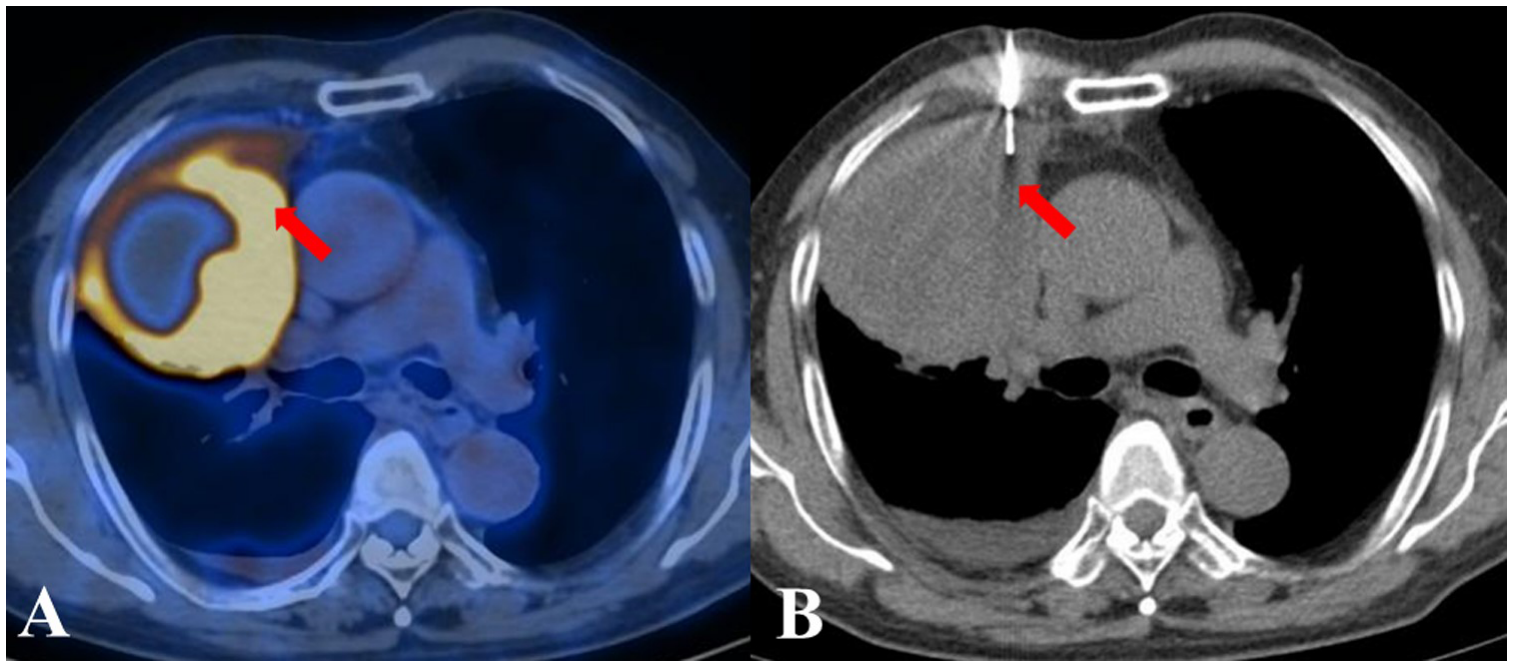
A patient from the PET/CT group displayed inhomogeneous metabolism in a suspected lung lesion. By referring to the PET/CT image displayed side by side, FDG-avid tissue was successfully located by the needle. The biopsy pathological examination considered squamous cell carcinoma, which was subsequently corroborated during follow-up. **(A)** The previous PET/CT images. **(B)** CT-guided TTNB.

**Figure 3:**
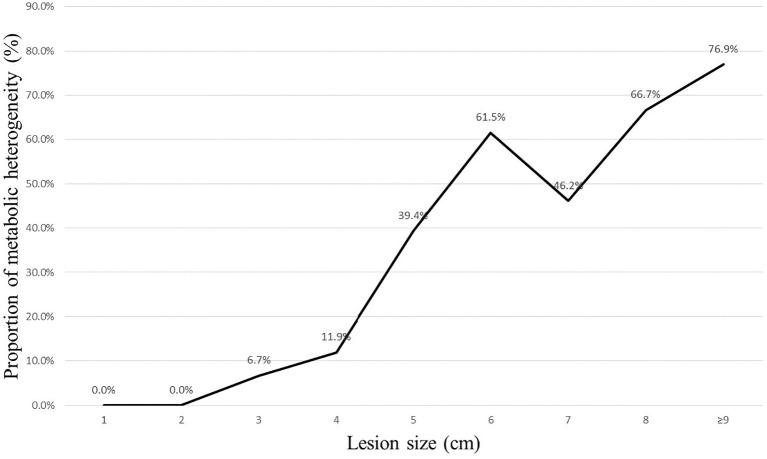
Relationship between proportion of metabolic heterogeneity and lesion size.

After splitting the results in each group into two classes, as detailed in **Tables 3** and **4**, the PET/CT group had 211 patients classified as diagnostic success (211/227, 93.0%) and 16 patients classified as diagnostic failure (16/227, 7.1%; including 8 cases of non-diagnostic and 8 cases of FN). In contrast, the CT group had 192 (192/231, 83.1%) patients classified as diagnostic success and 39 patients classified as diagnostic failure (39/231, 16.9%; including 22 cases of non-diagnostic and 17 cases of FN). When compared to the CT group, the PET/CT group showed a higher likelihood of successful diagnosis for TTNB (OR, 2.68; 95CI%, 1.45 - 4.95) with significant differences between the two groups (P = 0.001). The subgroup analysis further revealed that this advantage was observed in both malignant (OR, 2.25; 95CI%, 1.14 - 4.45) and benign lesions (OR, 4.90; 95CI%, 1.02 - 23.53), and the effect was not influenced by the nature of the lesion (P for interaction = 0.375). When the lesion size was analyzed hierarchically by 3 cm, the PET/CT group demonstrated superiority in large lesions (OR, 4.81; 95CI%, 2.03 - 11.36) (**Figures 1**, **2**), while showing moderate effect in small lesions (OR, 1.09; 95CI%, 0.42 - 2.81). Significant effect modification was observed in large lesions for TTNB in the PET/CT group (P for interaction = 0.023). Regarding the cause for effect modification in the PET/CT group, lesions larger than 3 cm were found to correlate with an inhomogeneous fludeoxyglucose uptake pattern. **Figure 3** showed a positive correlation between lesion size and the proportion of metabolic heterogeneity. The proportion of metabolic heterogeneity was higher for lesions larger than 3 cm than for lesions smaller than 3 cm (chi-square test, P < 0.001). In other subgroup analyses, although the PET/CT group had higher diagnostic efficacy in detecting single lesion (OR, 3.07; 95CI%, 1.44 - 6.54), specimens with ≥ 3 lesions (OR, 2.64; 95CI%, 1.26 - 5.52), and lesions in the upper and middle lobes (OR, 3.78; 95CI%, 1.35 - 10.55), there was no statistically significant differences compared to multiple lesions (P for interaction = 0.519), specimens with ≤ 2 lesions (P for interaction = 0.966), and lesions in the lower lobe (P for interaction = 0.399).

**Table 3:** Subgroup analysis between the two groups, according to final diagnosis, lesion size, number of lesions, number of specimens, and Lobar location.

Subgroup	PET/CT group		CT group		OR (95% CI)		P	P for interaction
	Success	Failure	Success	Failure				
Overall	211	16	192	39	2.68 (1.45 - 4.95)	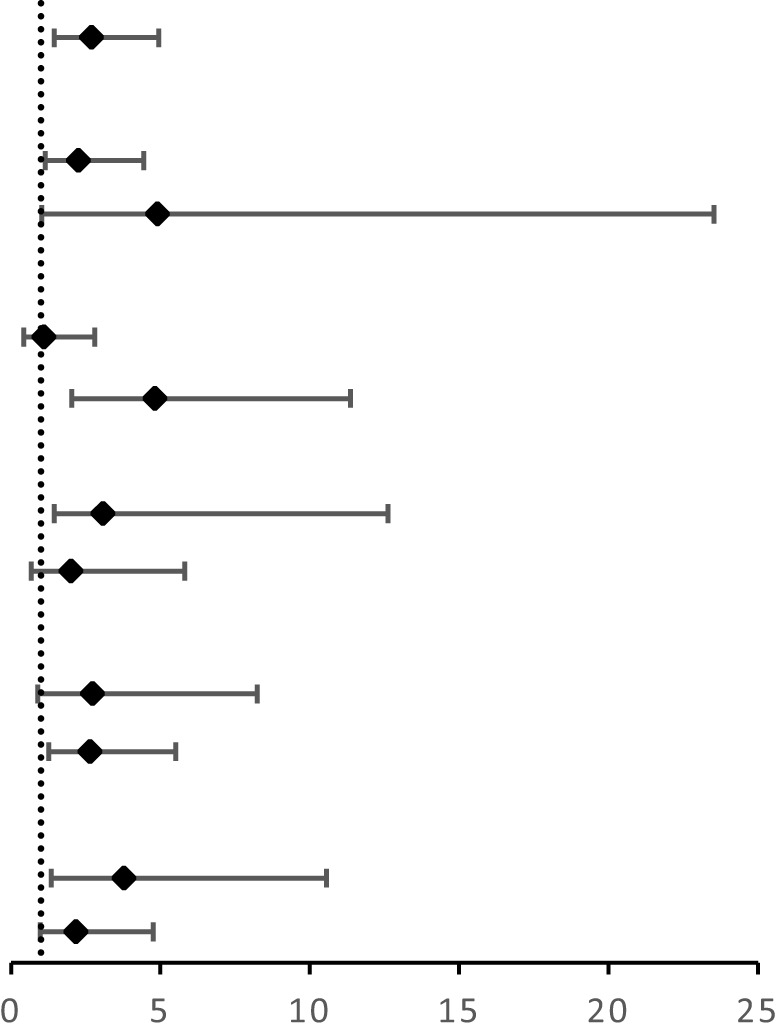	0.001	
Final diagnosis							
Malignant	180	14	154	27	2.25 (1.14 - 4.45)		0.017	0.375
Benign	31	2	38	12	4.90 (1.02 - 23.53)		0.033	
Lesion size (minimum diameter)							
≤3 cm	56	9	63	11	1.09 (0.42 - 2.81)		0.864	0.023
>3 cm	155	7	129	28	4.81 (2.03 - 11.36)		<0.001	
Number of lesions							
Single	144	10	136	29	3.07 (1.44 - 6.54)		0.003	0.519
Multiple	67	6	56	10	1.99 (0.68 - 5.82)		0.201	
Number of specimens							
≤2	47	5	45	13	2.72 (0.90 - 8.24)		0.070	0.966
≥3	164	11	147	26	2.64 (1.26 - 5.52)		0.008	
Lobar location							
U and M	131	5	118	17	3.78 (1.35 - 10.55)		0.007	0.399
L	80	11	74	22	2.16 (0.98 - 4.76)		0.052	

U, upper lobe; M, middle lobe; L, lower lobe.

**Table 4:** The reasons of diagnostic failure between the two groups.

Reasons	PET/CT group	CT group	Total
FN	8 (32.0%)	17 (68.0%)	25
Non-diagnosis			
Necrosis	1 (11.1%)	8 (88.9%)	9
Inadequate sampling	3 (27.3%)	8 (72.7%)	11
normal tissue	4 (40.0%)	6 (60.0%)	10

Complications occurred in 33 patients (14.5%) in the PET/CT group with 31 cases pneumothorax (13.7%), 1 case of hemothorax (0.4%), 1 case of pleural reaction (0.4%). In the CT group, 43 patients (18.6%) had complications with 42 cases pneumothorax (18.2%), 1 case of hemothorax (0.4%). No statistically significant difference was found between the two groups (P = 0.241). There were no grade ≥ 4 complications (Complication causing a permanent sequelae or death) occurred in either group according to CIRSE classification (15).

## 4 Discussion

Many studies have reported better diagnostic performance for PET/CT guided biopsy as it allows for targeting the highest metabolic area within the entire lesion (8, 9, 16). In our study, we found that the PET/CT group demonstrated higher diagnostic efficacy compared to the CT group. These findings were consistent with the results reported by Fontana et al. (9), further supporting the advantages of PET/CT-aided CT-guided TTNB. Specifically, the PET/CT group showed a significant improvement in sensitivity and diagnostic accuracy rate. When considering non-diagnostic cases, the overall diagnostic success rate increased markedly in PET/CT group (93.0% vs. 83.1%, P = 0.001).

However, selection bias often occurred, with the PET/CT group exhibiting a significantly higher proportion of malignant patients compared to the CT group (7, 12). This selection bias may act as a potential confounding factor, thereby diminishing result reliability. Moreover, Purandare et al. indicated PET/CT only provided an incremental value of 40.7% in lung biopsy (13), which means obtaining PET/CT scans before each lung biopsy may not be necessary or cost-effective. To this end, we explored subgroup analysis.

Laura et al. reported the performance of FDG-PET/CT in solitary pulmonary nodule based on pre-test likelihood of malignancy, they found that sensitivity and PPV were higher (P < 0.05) in intermediate and high-risk patients (11). This suggests that patients with high FDG uptake may be more likely to be recommended for biopsy, resulting in a higher rate of malignant lesions in the PET/CT group. Thus, Cerci et al. have hypothesized that selection bias may be related to the better accuracy of PET/CT in the evaluation of lung lesions, noticeably nodules with an intermediate likelihood of malignancy and presenting areas of hypermetabolism (7). Another recent study suggested that low fludeoxyglucose uptake (SUV max ≤ 2.5) may be one of the reasons for inconclusive histology following lung biopsy (17). In our research, only 6 (2.6%) patients of PET/CT group presented with low fludeoxyglucose uptake of lung lesion. The distribution of malignant and benign lesions also showed significant differences between the two groups (P = 0.048). Further hierarchical test revealed that the PET/CT group have a higher likelihood for diagnostic success in both malignant (OR, 2.25; 95CI%, 1.14 - 4.45) and benign lesions (OR, 4.90; 95CI%, 1.02 - 23.53), and this advantage was not influenced by the nature of the lesion (P for interaction = 0.375). After adjusting for this bias, our results (adjusted OR, 2.62; 95% CI, 1.41 - 4.86; P = 0.002) remained close to the initial results (unadjusted OR, 2.68; 95% CI, 1.45 - 4.95, P = 0.001). Therefore, the final diagnosis (malignant or benign) cannot be regarded as a confounding factor in our research. In practice, even if the nature of the lesion may affect the success of the biopsy, it remains difficult to guide clinical practice because the final diagnosis cannot be known before conducting TTNB.

Lesion size is a crucial factor that affects the diagnostic accuracy of lung biopsy. Yeow et al. concluded lung lesions smaller than 1.5 cm (which pose technical difficulty) and larger than 5 cm (which are associated with a higher necrosis rate) affect diagnostic accuracy of CT-guided TTNB. In their study, the necrosis rate began to rise rapidly when the lung lesions were larger than 3 cm, and exceeded 10% when the lung lesions were larger than 5 cm (18). Hiraki et al. reported all their false negative results in lesions ≥ 3.1 cm were attributed to the presence of necrosis (3). Thus, we set 3 cm as a cut-off point for subgroup analysis. The results showed that the PET/CT group had significantly higher diagnostic efficacy when the lesion was larger than 3 cm, while showing moderate effect when the lesion was smaller than 3 cm (P for interaction = 0.023). Similarly, Lin et al. indicated that CT-guided biopsy with prior PET/CT fusion imaging is particularly helpful in improving diagnostic yield and accurate rate of biopsy in lung masses (diameter larger than 3 cm) (19). Specifically, large lesions may contain inflammatory tissue or necrosis, which could increase the risk of diagnostic failure (10, 18). Erdoğan et al. discovered that the Metabolic Tumor Volume, which was calculated by region of interest replacement in metabolically active area in each slice, showed a direct proportional increase with the diameter of the solitary pulmonary nodule (SPN) (20). However, when they divided the SPNs into two groups based on diameter, such as < 2 cm and ≥ 2 cm, this positive correlation became unstable (evaluated using Spearman’s Rho test, R value of 0.582 vs. 0.463), which means more non-neoplastic cells emerge in some larger SPNs. Piacentino et al. demonstrated PET/CT can help identify the hypermetabolic areas within large lesions, with the SUVmax area showed higher percentages of neoplastic cells. Conversely, the SUVmin area expressed more inflammation, fibrosis, necrosis, and normal lung parenchyma (10), specimens obtained in this situation may be inadequate for a conclusive histopathological evaluation. Herein, sixteen patients encountered diagnostic failure in CT group, with 8 cases (8/9, 88.9%) of necrosis and 8 cases (8/11, 72.7%) of inadequate sampling. On the other hand, technical difficulty of small lesions decreased the diagnostic success rate in our study, whether with or without metabolic information, meaning the enhanced value of PET/CT in small lesions for TTNB was limited. Four normal tissue (4/10, 40%) from the PET/CT group led to non-diagnosis, and both of these lesions were smaller than 1.5 cm. In addition, a positive correlation between lesion size and the proportion of metabolic heterogeneity was found in our study, which could explain the effect modification in the PET/CT group.

In theory, PET/CT has the potential to guide the selection of multiple lesions for biopsy (21), thus improving the success rate of diagnosis. However, our research showed that PET/CT did not provide added value for TTNB of multiple lung lesions. One reason might be attributed to the presence of numerous ground glass nodules in the multiple lung lesions biopsy in our study, which usually could not significantly interfere with the selection of puncture sites. A study involving 73 patients with multifocal pure ground glass nodules demonstrated that whole-body PET/CT did not offer definitive benefits compared to chest CT alone (22). Besides, some patients with multiple lung lesions exhibited large differences in lesion size, which could not interfere with the selection of puncture sites in clinical practice.

The complication rate was found to be 14.5% in the PET/CT group and 18.6% in the CT group, indicating no statistically significant difference between the two groups (P = 0.100). These findings were similar to those of previous studies (7).

Despite the superiority of PET/CT in detecting large lesions, eight false negative results in the PET/CT group were inevitable in our study. Because hypermetabolic activity can also be observed in some benign lesions (21), such as acute inflammation or tuberculosis, making it difficult to accurately distinguish where neoplastic cells are present. Moreover, respiratory motion during the procedure may cause the needle tip to fail to locate to the predetermined spot, particularly in the lower lobe.

## 5 Conclusion

^18^F-FDG-PET/CT enhances the diagnostic efficacy of CT-guided transthoracic needle biopsy for lung lesions, and the incremental value can be modified by lesion size, particularly when the diameter is larger than 3 cm.

## Data availability statement

The original contributions presented in the study are included in the article/**Supplementary Material**. Further inquiries can be directed to the corresponding authors.

## Ethics statement

The studies involving human participants were reviewed and approved by the Ethics committee of the China-Japan Union Hospital of Jilin University. Written informed consent from the patients/ participants was not required to participate in this study in accordance with the national legislation and the institutional requirements.

## Author contributions

WL: Conceptualization, Investigation, Methodology, Validation, Writing – original draft, Writing – review & editing:. LB: Conceptualization, Project administration, Supervision, Validation, Writing – review & editing. SG: Conceptualization, Resources, Software, Validation, Writing – review & editing. BJ: Methodology, Validation, Visualization, Writing – review & editing.

## References

[1] Sung H Ferlay J Siegel RL Laversanne M Soerjomataram I Jemal A. Global cancer statistics 2020: globocan estimates of incidence and mortality worldwide for 36 cancers in 185 countries. CA Cancer J Clin (2021) 71:209–49. doi: 10.3322/caac.21660 33538338

[2] Bironzo P Di Maio M. A review of guidelines for lung cancer. J Thorac Dis. (2018) 10:S1556–63. doi: 10.21037/jtd.2018.03.54 PMC5994504 29951306

[3] Hiraki T Mimura H Gobara H Iguchi T Fujiwara H Sakurai J. Ct fluoroscopy-guided biopsy of 1,000 pulmonary lesions performed with 20-gauge coaxial cutting needles: diagnostic yield and risk factors for diagnostic failure. Chest (2009) 136:1612–7. doi: 10.1378/chest.09-0370 19429718

[4] Anzidei M Porfiri A Andrani F Di Martino M Saba L Catalano C. Imaging-guided chest biopsies: techniques and clinical results. Insights Imaging. (2017) 8:419–28. doi: 10.1007/s13244-017-0561-6 PMC5519500 28639114

[5] Lin CY Chang CC Chu CY Huang LT Chung TJ Liu YS. Computed tomography-guided transthoracic needle biopsy: predictors for diagnostic failure and tissue adequacy for molecular testing. Front Med (Lausanne). (2021) 8:650381. doi: 10.3389/fmed.2021.650381 34095167 PMC8169979

[6] Groheux D Quere G Blanc E Lemarignier C Vercellino L de Margerie-Mellon C. Fdg pet-ct for solitary pulmonary nodule and lung cancer: literature review. Diagn Interv Imaging. (2016) 97:1003–17. doi: 10.1016/j.diii.2016.06.020 27567555

[7] Cerci JJ Bogoni M Cerci RJ Masukawa M Neto C Krauzer C. Pet/ct-guided biopsy of suspected lung lesions requires less rebiopsy than ct-guided biopsy due to inconclusive results. J Nucl Med (2021) 62:1057–61. doi: 10.2967/jnumed.120.252403 33384323

[8] Guralnik L Rozenberg R Frenkel A Israel O Keidar Z. Metabolic pet/ct-guided lung lesion biopsies: impact on diagnostic accuracy and rate of sampling error. J Nucl Med (2015) 56:518–22. doi: 10.2967/jnumed.113.131466 25698780

[9] Fontana F Piacentino F Ierardi AM Carrafiello G Coppola A Muollo A. Comparison between cbct and fusion pet/ct-cbct guidance for lung biopsies. Cardiovasc Intervent Radiol (2021) 44:73–9. doi: 10.1007/s00270-020-02613-3 32895781

[10] Piacentino F Fontana F Zorzetto G Saccomanno A Casagrande S Franzi F. Could maximum suv be used as imaging guidance in large lung lesions biopsies? Double sampling under pet-ct/xperguide fusion imaging in inhomogeneous lung uptaking lesions to show that it can make a difference. Technol Cancer Res Treat (2023) 22:2081080900. doi: 10.1177/15330338221144508 PMC10155026 37116886

[11] Evangelista L Cuocolo A Pace L Mansi L Del VS Miletto P. Performance of fdg-pet/ct in solitary pulmonary nodule based on pre-test likelihood of Malignancy: results from the italian retrospective multicenter trial. Eur J Nucl Med Mol Imaging. (2018) 45:1898–907. doi: 10.1007/s00259-018-4016-1 29736699

[12] Tezcan MA Ozsoy IE Karacavus S Karaman H. Accuracy of metabolic imaging-guided transthoracic biopsy in lung cancer. J Coll Physicians Surg Pak (2022) 32:152–6. doi: 10.29271/jcpsp.2022.02.152 35108782

[13] Purandare NC Kulkarni AV Kulkarni SS Roy D Agrawal A Shah S. 18f-fdg pet/ct-directed biopsy: does it offer incremental benefit? Nucl Med Commun (2013) 34:203–10. doi: 10.1097/MNM.0b013e32835c5a57 23353885

[14] Yoon SH Lee SM Park CH Lee JH Kim H Chae KJ. 2020 Clinical practice guideline for percutaneous transthoracic needle biopsy of pulmonary lesions: a consensus statement and recommendations of the Korean society of thoracic radiology. Korean J Radiol (2021) 22:263–80. doi: 10.3348/kjr.2020.0137 PMC7817630 33236542

[15] Filippiadis DK Binkert C Pellerin O Hoffmann RT Krajina A Pereira PL. Cirse quality assurance document and standards for classification of complications: the cirse classification system. Cardiovasc Intervent Radiol (2017) 40:1141–6. doi: 10.1007/s00270-017-1703-4 28584945

[16] Intepe YS Metin B Sahin S Kaya B Okur A. Our transthoracic biopsy practices accompanied by the imaging process: the contribution of positron emission tomography usage to accurate diagnosis. Acta Clin Belg. (2016) 71:214–20. doi: 10.1080/17843286.2016.1155810 27142092

[17] Tipaldi MA Ronconi E Krokidis ME Zolovkins A Orgera G Laurino F. Diagnostic yield of ct-guided lung biopsies: how can we limit negative sampling? Br J Radiol (2022) 95:20210434. doi: 10.1259/bjr.20210434 34808070 PMC8822563

[18] Yeow KM Tsay PK Cheung YC Lui KW Pan KT Chou AS. Factors affecting diagnostic accuracy of ct-guided coaxial cutting needle lung biopsy: retrospective analysis of 631 procedures. J Vasc Interv Radiol (2003) 14:581–8. doi: 10.1097/01.rvi.0000071087.76348.c7 12761311

[19] Lin Y Xu Y Lin J Fu L Sun H Huang Z. Improving ct-guided transthoracic biopsy diagnostic yield of lung masses using intraprocedural ct and prior pet/ct fusion imaging. BMC Pulm Med (2022) 22:311. doi: 10.1186/s12890-022-02108-6 35964027 PMC9375328

[20] Erdogan M Evrimler S Aydin H Karaibrahimoglu A Sengul SS. Solitary pulmonary nodule: morphological effects on metabolic activity assessment. Mol Imaging Radionucl Ther (2019) 28:112–9. doi: 10.4274/mirt.galenos.2019.65707 PMC6746010 31507144

[21] Cerci JJ Tabacchi E Bogoni M. Fluorodeoxyglucose-pet/computed tomography-guided biopsy. Pet Clin (2016) 11:57–64. doi: 10.1016/j.cpet.2015.08.001 26590444

[22] Li M Wan Y Zhang L Zhou LN Shi Z Zhang R. Synchronous multiple lung cancers presenting as multifocal pure ground glass nodules: are whole-body positron emission tomography/computed tomography and brain enhanced magnetic resonance imaging necessary? Transl Lung Cancer Res (2019) 8:649–57. doi: 10.21037/tlcr.2019.09.10 PMC6835114 31737500

